# Modelling the Self-Assembly of Elastomeric Proteins Provides Insights into the Evolution of Their Domain Architectures

**DOI:** 10.1371/journal.pcbi.1002406

**Published:** 2012-03-01

**Authors:** Hongyan Song, John Parkinson

**Affiliations:** 1 Program in Molecular Structure and Function, Hospital for Sick Children, Toronto, Ontario, Canada; 2 Departments of Biochemistry and Molecular Genetics, University of Toronto, Toronto, Ontario, Canada; Stockholm University, Sweden

## Abstract

Elastomeric proteins have evolved independently multiple times through evolution. Produced as monomers, they self-assemble into polymeric structures that impart properties of stretch and recoil. They are composed of an alternating domain architecture of elastomeric domains interspersed with cross-linking elements. While the former provide the elasticity as well as help drive the assembly process, the latter serve to stabilise the polymer. Changes in the number and arrangement of the elastomeric and cross-linking regions have been shown to significantly impact their assembly and mechanical properties. However, to date, such studies are relatively limited. Here we present a theoretical study that examines the impact of domain architecture on polymer assembly and integrity. At the core of this study is a novel simulation environment that uses a model of diffusion limited aggregation to simulate the self-assembly of rod-like particles with alternating domain architectures. Applying the model to different domain architectures, we generate a variety of aggregates which are subsequently analysed by graph-theoretic metrics to predict their structural integrity. Our results show that the relative length and number of elastomeric and cross-linking domains can significantly impact the morphology and structural integrity of the resultant polymeric structure. For example, the most highly connected polymers were those constructed from asymmetric rods consisting of relatively large cross-linking elements interspersed with smaller elastomeric domains. In addition to providing insights into the evolution of elastomeric proteins, simulations such as those presented here may prove valuable for the tuneable design of new molecules that may be exploited as useful biomaterials.

## Author Summary

Elastomeric proteins such as elastin, resilin, abductin and wheat gluten represent a remarkable class of self-assembling proteins that provide properties of extensibility and elastic recoil. Although unrelated from an evolutionary viewpoint, these proteins nonetheless share a common sequence design involving highly repetitive elastomeric regions interspersed with elements capable of forming cross-links that help stabilize the formation of polymers. Attempts to explore the influence of domain architecture on the self-assembly and mechanical properties of elastomeric proteins at the molecular level have largely been hindered by a general lack of detailed structural information. Here we introduce a novel theoretical study based on random walks to simulate the self-assembly of elastomeric proteins. Applying this model, we explored the impact of different configurations of elastomeric and cross-linking elements on the stability of the resultant polymer. Through exploring the complex relationships between elastomeric domains, required to drive self-assembly, and cross-linking domains, required for structural integrity, results from these simulations provide insights into the molecular basis for the evolution of elastomeric proteins as well as help guide the rational design of novel elastomeric-peptides.

## Introduction

Elastomeric proteins such as elastin, resilin, abductin, spider dragline silks and wheat gluten represent a remarkable class of self-assembling proteins that provide properties of extensibility and elastic recoil [1]. Consequently there has been much interest in their development as mechanically active biomaterials for purposes of tissue replacement and tissue engineering 2–4. In addition to their elastomeric properties, their ability to self-assemble suggests a role as scaffolds for tissue engineering with tremendous promise in regenerative medicine [5]. Of particular interest has been the synthesis of recombinant peptides that in addition to capturing the functional properties of the full length protein, may be readily modified to include e.g. cell recognition sites or other bioactive domains providing additional benefits for tissue engineering applications [6]–[10]. Intriguingly, each protein appears to have arisen independently: elastin is a vertebrate protein that plays an integral role in the extracellular matrix (ECM) of elastic tissues such as aorta, arteries and lung parenchyma; resilin is found in specialized regions of the cuticle of insects where it functions as an energy store; abductin is located in the hinge region of bivalves, responsible for opening the shell upon relaxation of the abductor muscle; spider dragline silks are used by spiders as safety lines or for constructing the frames of their webs; and gluten is a plant protein that may have evolved to facilitate efficient packaging as a food store for developing seedlings [11]. Although unrelated from an evolutionary viewpoint, these proteins nonetheless share a common sequence design involving highly repetitive often hydrophobic regions (which we term elastomeric domains) interspersed with elements capable of forming cross-links (which we term cross-linking domains) that help stabilize the formation of homopolymers [6], [12]. For example, elastin is largely comprised of alternating hydrophobic domains rich in VPGVG repeats, and domains containing KAA(A)K motifs that allow the formation of desmosine based cross-links. Each domain tends to be associated with a single exon. Abductin, gluten and resilin are similarly largely composed of repetitive hydrophobic elements interspersed with tyrosine residues, involved in the formation of di- and tri-tyrosine cross links [8], [13], [14]. Dragline spider silks are slightly different with elastomeric domains that often include glutamine interspersed with alanine-rich domains which are thought to derive cross-links through non-covalent interactions [15]. It is thought that elastomeric domains are relatively disordered; composed mainly of β-turns and β-strands [16]. It is further suggested that extension of these regions within an aqueous environment leads to an increase in local ordering of water molecules and that the remarkable resilience of these proteins arises from the increase in entropy associated with the subsequent relaxation of the domains into their relatively disordered states [17], [18].

In addition to imparting properties of elastic recoil, the hydrophobic regions of elastomeric proteins are thought to be responsible for driving their self-assembly at least for elastin [12], [19]. For example, while the incorporation of tropoelastin into the extracellular matrix occurs in a complex step-wise process [20], tropoelastin has also been shown to self-assemble *in vitro* in a reversible temperature-induced process termed coacervation, reliant on interactions between elastomeric domains [6], [21], [22]. These interactions control the alignment of the cross-linking domains, which subsequently form lysine based cross-links that stabilize the resultant polymeric matrix [23]–[25]. Using recombinant polypeptides based on human elastin, it has also been shown that as few as three elastomeric domains flanking two crosslinking domains are sufficient to support self-assembly [6], [26]. Furthermore, changes in the number and arrangement of the elastomeric and cross-linking regions can significantly impact their assembly and mechanical properties [6], [27], [28]. It is therefore striking to note that across vertebrates, elastin has evolved a range of different architectures [19]. For example, while human elastin is composed of 15 elastomeric domains interspersed with 14 cross linking domains (encoded by 18 and 14 exons respectively), zebrafish maintains two copies, both composed of 24 elastomeric domains interspersed with 24 cross linking domains (the domains in the first copy being encoded by 31 and 25 exons respectively, while those in the second copy are encoded by 35 and 24 exons respectively). Interestingly, the copies in zebrafish appear to be undergoing processes of sub-functionalization with discrete, albeit overlapping, patterns of tissue expression [29]. Despite the recent report of a structure of tropoelastin derived using small angle X-ray and neutron scattering [30], attempts to explore the influence of architecture on the self-assembly and mechanical properties of elastomeric proteins at the molecular level have largely been hindered by a general lack of detailed structural information. Typically the cross-linked polymers do not form resolvable crystals, while the use of classical spectroscopic techniques is precluded by the insoluble nature of the matrix [31]. As a result, studies exploring the functional consequences of changes in the arrangement of elastomeric and cross-linking elements have been limited.

For elastin, a number of molecular dynamic simulations have been performed in attempts to derive insights into the structural and biomechanical properties of elastin [32]–[36]. Although effective in the study of relatively small molecules (<100 atoms), statistical convergence for larger molecules is difficult to attain. Consequently, further investigations focusing on the relationships between the supramolecular organization of elastomeric proteins and their mechanical properties require the formulation of mesoscale methods. These methods have been exploited for a number of applications from viral coat and microtubule assembly to models of collagen fibrillogenesis [37]–[45]. Elastomeric proteins present a particularly suitable system for the application of such methods due to known constraints that can be imposed during the modeling process. These include the necessity to ensure that neighbouring elastomeric domains from two elastomeric molecules are juxtaposed such that cross-linking domains are aligned to permit the formation of cross-links that are critical to the biomechanical properties of the polymeric matrix.

Here we describe a theoretical study that applies a modified Diffusion-Limited Aggregation (DLA) [46], [47] algorithm to simulate the self-assembly of elastomeric proteins. DLA has previously been shown to be a useful model for a number of other biological processes including collagen fibrillogenesis and diatom morphogenesis [43], [45]. Applying this model, we performed a systematic investigation to examine the impact of different configurations of elastomeric and cross-linking elements on the morphology and stability of the assembled polymer. Through exploring the complex relationships between elastomeric domains, required to drive self-assembly, and cross-linking domains, required for structural integrity, results from these simulations provide insights into the molecular basis for the evolution of elastomeric proteins as well as help guide the rational design of novel elastomeric-peptides.

## Results/Discussion

### A modified DLA model of molecular aggregation that is driven through the minimization of exposed hydrophobic surface results in the formation of compact, fibre-like aggregates

**Figure 1:**
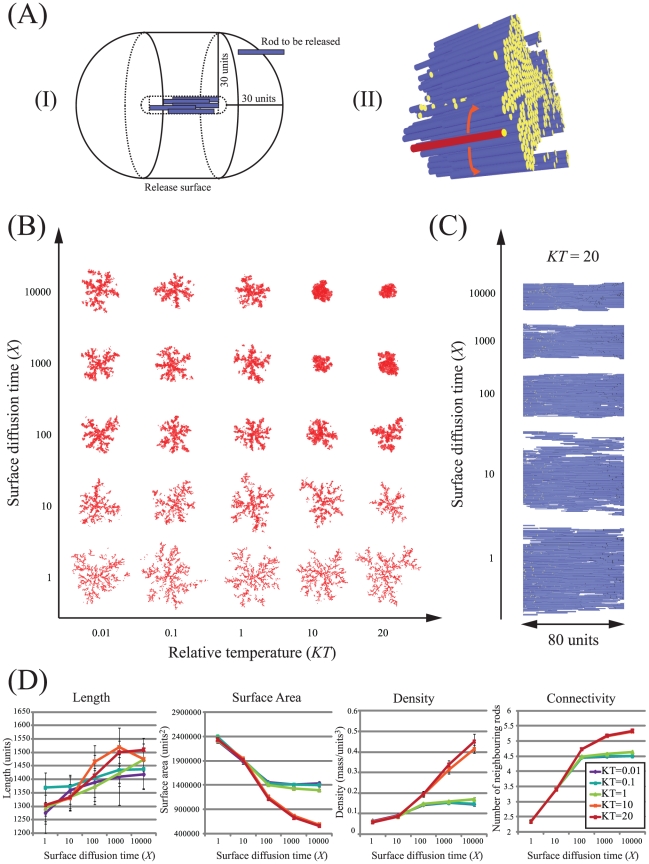
Impact of surface diffusion on aggregate morphology. (A) Implementation of modified DLA algorithm. (I) Schematic showing the release of new rods from a release template defined as 30 units distant from the growing aggregate. (II) View of a section of an aggregate generated with rods composed of a single elastomeric domain of length 20 units and diameter 1 unit, using *KT* = 20 and *X* = 1,000. Orange arrows indicate the direction of lateral surface diffusion for a newly accreted rod (red) that drive the minimization of exposed hydrophobic surface. (B) Phase diagram showing a two dimensional cross-section through a central 60 unit section of representative aggregates grown under different values of *KT* and *X*. (C) Side views of 80 units of the central section of representative fibrils from (B). (D) Quantitative measures of morphological characteristics of aggregates represented in the phase diagram (B). Aggregates were grown using 10,000 rods, standard deviations are from 10 replicates.

We devised a modified implementation of an off-lattice DLA model to simulate the self-assembly of elastomeric molecules, represented by circular rods composed of elastomeric and cross-linking domains combined into a variety of different architectures. Previous work on elastin polypeptides have shown that they self-assemble into biomaterials upon changes in temperature [6]. During this process, the polypeptides are effectively freely diffusing in solution (Brownian motion) prior to aggregation. Here, individual rods representing elastomeric protein monomers, are introduced and allowed to freely diffuse (reflecting the Brownian motion of molecules in solution [46], [47]) until they encounter the growing aggregate whereupon they adhere with a probability dependent upon the proportion of hydrophobic surface contact (see Materials and Methods). To further account for the free energy contributions of the elastomeric domains for driving self-assembly, once a rod has accreted to the growing aggregate it is assigned a probability of moving to an adjacent site depending upon the relative change in energy associated with exposed hydrophobic surface. Movement is restricted to the plane parallel to the long axis of the rod, i.e. the rod is allowed to ‘roll’ over the surface of the aggregate (Figure 1A). This was achieved by rotating the rod around the axis of a randomly selected adjacent neighbour. The number of such events is determined by a surface mobility term, *X*. which describes the number of potential ‘surface moves’ a particle may make after aggregating and essentially reflects the relative diffusion speed compared to the speed of aggregation. The probability of a particle moving into the new site was based on the Boltzmann function:
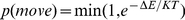
(1)where ΔE represents the change in energy calculated as the proportion of exposed hydrophobic surface before and after the proposed move. *KT* is a tunable temperature term, which effectively allows the particles to explore a greater number of conformations. This process is repeated several thousand times to construct large aggregates reminiscent of the fibrous-type structures associated with elastomeric fibrils [6], [7], [48]. Due to computational complexity, we did not allow for any intra-molecular flexibility, instead treating monomers as rigid particles. Consequently our model is not able to capture intermediate forms of assembly such as the droplets observed prior to the formation of elastin fibres [26], [49]. Nevertheless, even this simplified model allows us to explore the relationship between the molecular architecture of the elastomeric protein and its ability to assemble into organized arrays to generate fibres with distinct morphological and mechanical properties.

In an initial series of simulations we first explore the impact of surface diffusion on generating compact fibres through varying parameters *KT* and *X* in simulations of rods composed of a single elastomeric domain (Figure 1). At low values of *KT* and *X* the mobility of accreting rods is limited resulting in the generation of open, fractal-like aggregates (Figure 1B and C). Such aggregates are characterized as relatively poorly connected, short and of low density resulting in relatively high exposed surface area (Figure 1D). As *KT* and *X* increase, the surface diffusion process drives the generation of longer, denser and more highly connected aggregates. Studies of spider silk proteins and elastin polypeptides reveal fibres that are relatively compact and dense [6], [50], [51]. Thus to reflect these compact structures and also account for the influence of elastomeric domains on self-assembly, in subsequent experiments we chose values of *KT* and *X* that lead to the formation of relatively compact structures (20 and 1,000 respectively – NB a value of 10,000 for *X* was not used since a typical simulation using this value took ∼7 days on a single processor).

### Short-elastomeric domains interspersed with cross-linking elements reduce the potential for monomer misalignment

Elastomeric proteins are composed of alternating ‘domains’ of elastomeric and cross-linking elements. It has been demonstrated that elastin-like polypeptides with as few as five domains possess the ability to self-assemble [6], [12]. However, natural elastomeric proteins tend to be large – for example human elastin is composed of 29 alternating elastomeric - cross-linking domains while elastin from *Xenopus* can have as many as 48 domains [19]. Similarly, natural dragline spider silk proteins analysed to date also demonstrate very high molecular weights (250–320 kDa [52], [53]). Recent work on recombinant spider silk proteins, suggest that increasing the number of domains results in fibres with superior mechanical properties [51]. To explore these relationships further, we investigated the impact of introducing rods with alternating architectures and varying lengths into our simulations.

**Figure 2:**
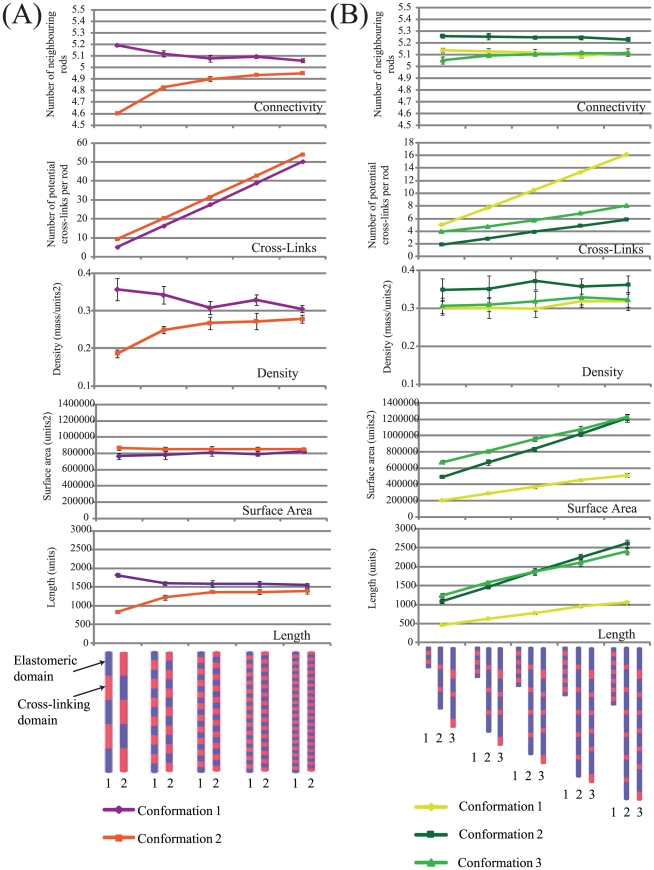
Quantitative effects of domain number on aggregate morphology and stability. (A) Graphs showing the impact of increasing the relative number of domains while keeping rod length constant (20 units). Two conformations were examined: conformation 1 refers to rods in which the number of elastomeric domains (blue) exceeds the number of cross-linking domains (red); conformation 2 refers to rods in which the number if cross-linking domains exceeds the number of elastomeric domains. (B) Graphs showing the impact of adding additional domains. Here three conformations were investigated: conformation 1 consists of rods composed of domains of length one unit; conformation 2 consists of rods composed of elastomeric domains of length 4 units and cross-linking domains of length 1 unit; and conformation 3 is an asymmetrical rod consisting of two sets of: a elastomeric domain of length five units and a cross-linking domain of length two, between which are increasing numbers of elastomeric domains of length four and a cross-linking domain of length one. Error bars indicate standard deviations for ten replicates.

First we examined the ability of rods composed of a simple alternating architecture of three elastomeric domains with two cross-linking elements to form ordered arrays (Figure S1). In these and subsequent simulations only elastomeric domains are considered for assembly and surface-diffusion purposes, while the cross-linking domains were assessed for their ability to form cross-links. Qualitatively, the use of the alternating architecture resulted in the generation of similar morphologies as observed for the rods composed of a single elastomeric domain; increases in *KT* and *X* results in the generation of more compact aggregates (Figure S1B and C). We did note an increase in aggregate length with the use of the alternating domain architecture. For example at *KT* = 20 and *X* = 1,000, the length of the aggregate using the alternating architecture was 1782+/−82 units compared to 1509+/−43 for aggregates generated from rods composed only of a single domain. This is presumably related to optimizing the alignment between neighbouring elastomeric domains. Such alignment leads to overlap between cross-linking domains and allows the prediction of aggregate stability (see Materials and Methods). Currently little is known about the order of cross-linking, although studies on recombinant tropoelastin have suggested that coacervation (self-assembly) is required to generate cross-links among recombinant polypeptides [6], [20]. Here we simply assign a potential cross-link if two cross-linking domains on neighbouring rods overlap by at least 50% of their respective lengths. As expected more compact aggregates have a larger number of potential cross-links. Next we were interested in examining the effect of increasing the number of domains on aggregate morphology and stability. In a first set of experiments, we increased the number of domains while maintaining a constant rod length. Two types of alternating architectures were examined in which either the elastomeric or the cross-linking domains were placed at the rod termini (conformation 1 and 2 respectively, see Figure 2A). Trivially, increasing the number of domains resulted in an increase in the number of potential cross-links. Of greater interest, increasing the relative proportion of elastomeric to cross-linking domains led to an increase in aggregate length and density; surface area was unaffected. The evolutionary implications of these findings are presented in conclusions.

Next we examined the impact of increasing rod length through the addition of extra alternating domains (Figure 2B). Three groups of domain architecture, with different lengths of cross-linking and elastomeric domains were investigated (designated conformation 1–3). As expected for all three conformations explored, an increase in domains resulted in an increase in cross-links, length and surface area. Consistent with this, the rods with the highest proportion of elastomeric domains (conformation 2–12 units of elastomeric domain: 2 units of cross-linking domain), resulted in the highest density. Furthermore, the addition of extra domains resulted in an increase in cross-links for all conformations. However, despite sharing the same number of cross-linking domains as conformation 2 and fewer such domains than conformation 3, aggregates generated from conformation 1 rods resulted in considerably more potential cross-links. Interestingly, aggregates generated from conformation 2 had consistently fewer cross-links compared to the other two conformations, despite appearing to possess more neighbours (higher connectivity). This suggests that the lack of cross-links in these aggregates is due to misalignment of cross-linking domains between neighbouring rods. Hence minimizing the size of the elastomeric domain, reduces the potential for misalignment. This finding suggests a potential driving force for maintaining relatively short elastomeric domains associated with these proteins. In elastin, for example, individual elastomeric domains may be as short as 5 amino acids, as found between the two cross-linking domains encoded by exons 23 and 24 in tropoelastin-2 of *Xenopus tropicalis* [54]. However, it is noted that here we only explored a limited number of conformations, in the next section, we explore the impact of a wider range of rod architectures on aggregate morphology.

### Aggregates generated from rods with asymmetrical architectures and relatively small elastomeric domains are more resilient than their symmetrical counterparts

In addition to the number of domains, we were interested in exploring how the relative size of the two types of domain as well as their spacing may impact aggregate morphology and stability. We therefore surveyed 32 different domain architectures, ranging from rods composed of as few as three domains to those composed of six domains (designated architectures 1–32 - Figure S2). All domain architectures resulted in the generation of aggregates. However, while the number of connections between neighbouring rods was relatively constant, aggregate morphology (as measured by length and surface area) and stability (as measured by density) varied considerably between architectures. Longest aggregates were generated by rods composed of large cross-linking domains with two relatively small elastomeric domains at the termini (architectures 18, 19, 23 and 24). This is presumably due to minimizing the overlap between accreting rods; the two architectures with additional elastomeric domains in the center of the rods were slightly shorter. Densest aggregates were generated by rods composed of five symmetrically spaced domains, with three elastomeric domains encompassing two cross-linking domains (architectures 5, 16 and 22). Conversely, among the shortest and least dense aggregates were those composed from rods with five symmetrically spaced domains, each possessing three cross-linking domains encompassing two elastomeric domains (architectures 25, 28 and 29). A cross-sectional view of the architecture 28 aggregate reveals that rather than being relatively diffuse, the aggregate is composed of several relatively dense clusters of rods separated by large gaps in the overall structure (Figure S2). These dense local clusters likely occur through the perfect lateral alignment of neighbouring rods driven by the relatively small size of the elastomeric domains. At the same time, rods will occasionally accrete in a manner that generates a stagger. This stagger then results in the steric hindrance of further rods adding to the previously compact cluster.

**Figure 3:**
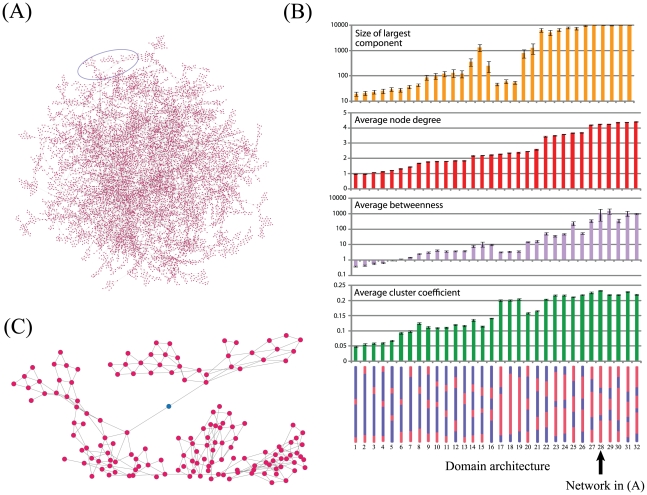
Network analysis of aggregate stability. Based on potential cross-links formed by neighbouring cross-linking domains, a network of rod connectivity can be generated (A). In this network nodes indicate individual rods and edges represent potential cross-links. (B) Graphs showing graph theoretical properties of networks generated for 32 different rod architectures composed of different numbers and sizes of elastomeric (blue) and cross-linking (red) domains. Domain architectures are indicated at the bottom. The arrow indicates architecture 28 used to construct the network in (A). Error bars indicate standard deviations for ten replicates. (C) Magnified section of the network presented in (A) highlighting a node (green) which has a high value of betweenness and low node degree and may therefore represent a weak point within the aggregate.

To gain deeper insights into the relationships between rod architecture and the structural integrity of resultant aggregates, we identified potential cross-links between neighbouring rods and used these data to construct networks in which nodes represent individual rods and edges represent potential cross-links (Figure 3A). The structural integrity of these networks can then be assessed through traditional graph based metrics that examine the networks tolerance for errors [55]. We therefore define the structural integrity of the network (and hence the aggregate from which it was derived) as its ability to maintain its connectivity through random removal of nodes within the network. Several metrics based on graph theory have previously been applied to derive quantitative measures of their structural integrity [56]. Among the more useful are measures of connectivity and centrality [55], [57], [58]. Here we apply four metrics: (1) size of the largest connected component. Some aggregates generate networks in which not all the nodes are connected, creating several smaller networks. The largest connected component indicates the number of nodes that together form the largest network. (2) average node degree, this is simply the average number of links per node in the network. (3) average betweenness which provides a measure of the distribution of load within the network. The betweenness of a node is typically calculated as the number of all shortest paths between all nodes in the network that go through that node, where a shortest path is the minimum number of links that connect any two nodes in the network. (4) average cluster coefficient which provides a measure of the degree to which nodes in a graph tend to cluster together. The cluster coefficient of a node is typically obtained from the number of interconnections between its neighbouring nodes as a fraction of all possible connections that those nodes could form. Of the 32 architectures examined here, only five resulted in aggregates that could be connected into a single entity through cross-links (architectures 28–32). These are all characterized as having three cross-linking domains with either two (architectures 28–30) or three (architectures 31 and 32) relatively small elastomeric domains. Furthermore, each of these aggregates have high average node degrees, betweenness and cluster coefficients, demonstrating the increased ability of these aggregates to form cross-links. Given the reduction in average connectivity of these aggregates (Figure S2), it appears that this ability likely arises from the interplay between the relatively small elastomeric domains driving the assembly of a regular ordered array of rods, together with the larger size of the cross-linking domains. While the former helps cross-linking domains to align, the latter increases the probability that there is sufficient overlap between neighbouring cross-linking domains to form a cross-link. However not all of these types of architectures form such well connected aggregates. For example, we note that three architectures composed of either a single or two relatively large cross-linking domains (architectures 17–19), possess relatively large cluster coefficients but nonetheless do not form a single large connected component. These architectures are therefore likely to be composed of relatively isolated groups of locally well connected rods, which are not globally well connected.

**Figure 4:**
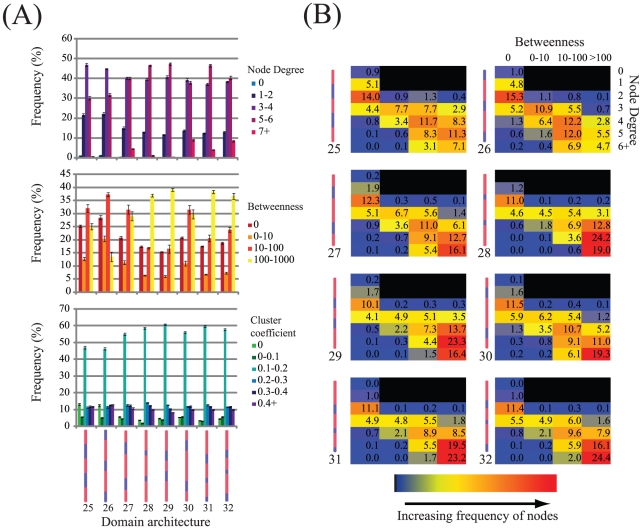
Detailed network statistics for aggregates generated from a select set of eight rod architectures. (A) Graphs showing distributions of node degree, betweenness and cluster coefficients for nodes generated from networks associated with the eight rod architectures leading to aggregates with the highest average node degree (domain architectures 25–32 in Figure 3). (B) Heatmaps showing the frequency of nodes (as a percentage) with specific values of betweenness and node degree. Rod architectures are indicated to the left of each heatmap. Aggregates composed of a large fraction of nodes which are both of high node degree and high betweenness are expected to be more resistant to mechanical failure. Standard deviations are provided for ten replicates.

These latter findings suggest that in addition to examining the average topological properties of a network, it may be important to examine the distribution of these properties. In previous work, we applied local rules of damage accumulation to model the ability of an aggregate to withstand stress [42]. However, here we assume that the distribution of topological properties may serve as a further measure of the ability of a particular network to withstand stress. For example, links associated with individual nodes that are not well connected but are relatively central to the network will be more likely to break (Figure 3C). Consequently, we may expect that aggregates which display relatively even distributions of connectivities would be more resilient than those with more extreme distributions. We therefore examined the distribution of three key metrics within the network for the eight architectures that result in the generation of aggregates with the highest average node degree (Figure 4A). Of the eight, aggregates composed of rod architectures 25 and 26 had fewer nodes that were well connected (node degree >4) and/or were very central to the network (betweenness >100). Furthermore, these two architectures display different distributions of cluster coefficients, with a larger proportion (>10%) of nodes having a value of 0. As noted from Figure S2, such distributions are associated with both aggregates having smaller, largest components compared with the remaining six architectures.

Of the remaining architectures, 28 and 29 were notable in having fewer nodes with seven or more connections, while architectures 27 and 30 had relatively fewer nodes of high betweenness (>100). Only aggregates composed of the asymmetrical rod architectures 31 and 32 had consistently large numbers of nodes with either high node degrees (>6) or betweenness (>100). These results indicate that these latter two architectures form aggregates that may best withstand stress. However, as noted above, rather than individual topological properties contributing to aggregate resilience, the combination of such properties are likely to play a more important role, i.e. rods which have high betweenness values but are well connected to other rods, are more resistant to applied stress. Figure 4B shows the distribution of different combinations of node degree and betweenness for the eight architectures. From these, we observe that aggregates composed of architectures 31 and 32 have a greater proportion of nodes (23.2% and 24.4% respectively) with six or more connections and high betweenness values (>100) compared with the other architectures. For architectures 27–30, while the proportion of such nodes is similar, we note that architectures 28 and 29 have considerably more nodes with five connections and betweenness >100 (24.2% and 23.3% respectively) compared with architectures 27 and 30 (12.7% and 11% respectively). Together these results suggest that aggregates composed of the asymmetrical architectures 31 and 32, with three relatively small elastomeric domains are likely to be more resilient to applied stress than their symmetrical counterparts (e.g. architectures 23, 24 and 26).

### Conclusions

In this study we sought to construct a simulation environment that can help us understand the impact of domain architecture on the morphology and structural integrity of polymers composed of elastomeric proteins. As we have shown here, the relative size of these domains may have a profound influence on the ability of these molecules to correctly align their cross-linking domains to help stabilize the resulting polymeric matrix. One relevant finding was that increasing the number of domains had little impact on aggregate morphology, but allowed the formation of additional cross-links. Elastomeric proteins have evolved multiple times independently within the eukaryotic lineage. Though they differ greatly in their structure and mechanical properties, they have converged on a distinctive sequence ‘style’, composed of alternating elastomeric and cross-linking domains. Interestingly, since arising in the vertebrate lineage, no short forms of elastin have been identified, varying from 15 elastomeric domains interspersed with 14 cross linking domains (encoded by 18 and 14 exons respectively) in human to 24 elastomeric domains interspersed with 24 cross linking domains (encoded by 35 and 24 exons respectively) in zebrafish tropoelastin-2 [19]. Similarly, natural dragline spider silk proteins analysed to date also demonstrate very high molecular weights (250–320 kDa [52], [53]).

While the origins of these proteins are still unclear, it appears that after their first appearance, there was a rapid period of evolution resulting in the formation of longer forms of these proteins. For elastin, many domains are encoded by a single exon, suggesting that for this protein at least, elongation of the alternating domain architecture occurred though duplications of pairs of elastomeric and cross-linking domain-encoding exons [19]. Results from our simulations, together with experimental data from mechanical studies investigating recombinant peptides based on spider silk proteins and elastin, suggest that this process of domain expansion may have been driven by improved mechanical properties [6], [51]. For example, Xia and co-workers applied stress-strain tests on polymer fibres generated from recombinant spider silk protein and found that those generated from 64-mer and 96-mer monomers are denser and stronger (higher breaking strain) than those generated from 16-mer and 32-mer monomer proteins [51]. Similarly, studies on recombinant tropoelastin also demonstrated improved mechanical performance from materials generated from longer recombinant polypeptides [6]. For example, polymers produced by human EP20-24^4^ (composed of human elastin exons 20,21,23,24,21,23,24,21,23,24,21,23,24 where exons 20 and 24 encode elastomeric domains and 21 and 23 encode cross-linking domains) have higher stress and strain at break (0.23±0.08 MPa and 103±24% respectively) than polymers produced by EP20-24-24 (composed of human elastin exons 20,21,23,24,21,23,24) (0.19±0.08 MPa and 86±42% respectively). From our simulations, these improved mechanical properties appear to have arisen through increasing the cross-linking capabilities of these proteins, while at the same time having little impact on overall fibre morphology.

A further prediction from our model is that smaller elastomeric domains are required to help reduce the potential for misalignment between cross-linking domains on neighbouring rods. For human elastin, we note that elastomeric domains show relatively variable sizes, from 25 amino acid residues (encoded by exons 28 and 29) up to 54 residues (encoded by exon 20). Furthermore, *Xenopus* and zebrafish contain copies of elastin that in addition to having more domains, also tend to have larger elastomeric domains (up to 146 residues as encoded with exon 46 of tropolastin 2 of zebrafish) [19]. While we expect the larger number of domains associated with elastin from *Xenopus* and zebrafish to increase the number of cross-links, we speculate that their larger size may be a necessary adaptation for driving hydrophobic interactions under cold-blooded conditions, as suggested previously [59]–[61], and/or may be responsible for a reduction in elastic modulus [29].

During this work, we note that more resilient aggregates form when the monomer is composed of relatively large cross-linking domains interspersed with relatively short elastomeric domains. Such patterns help align neighbouring rods and thereby increase the probability of forming cross-links. However, in these simulations, we did not take into account the absolute size of overlap required for a rod to accrete to the growing aggregate, i.e. in nature it is likely that a minimum size of hydrophobic interface is required to drive assembly. Nonetheless, this study highlights the potentially complex relationship between the relative size of the elastomeric domain required for self-assembly, and the impact of its size on the ability of the resultant aggregate to form cross-links. From a biomaterials perspective, to generate polymers with greatest cross-linking potential it may therefore be important to design monomers with relatively small domains that are highly hydrophobic. On a related note, an interesting finding was that asymmetric rods resulted in the most resilient aggregates. Again this suggests additional criteria that bioengineers may wish to take into consideration in the design of novel biomaterials.

It should be appreciated that in developing our modelling framework, we have made several assumptions. For example, we represent monomers as inflexible rods and introduce defined rules concerning the hydrophobic contribution to assembly as well as rules concerning the formation of cross-links. Such assumptions are likely to influence our findings and may not accurately reflect what occurs *in vivo*. We note, for example, a very recent structural study that investigated tropoelastin by small angle X-ray and neutron scattering, which suggests that while tropoelastin is an elongated molecule, it is asymmetric, possessing a “foot”-like structure at one end [30]. The authors of this study subsequently propose a head-to-tail model of assembly. While this would appear at odds with our own simulations, our study nonetheless allows an investigation into the role that domain configuration may have on monomer alignment and how this may propagate to morphological and structural properties for aggregates composed of 1000's of monomers. It is also worth noting that while tropoelastin monomers may not be well represented by inflexible rods, other elastomeric proteins such as abductin, resilin and gluten may be.

This study raises several testable hypotheses concerning the evolution and design of elastomeric polymers (e.g. shorter elastomeric domains are predicted to result in aggregates with more cross-links) that may be readily tested in experimental systems that focus on the design of novel biomaterials based on spider silk and elastin proteins [6], 50,51. Finally we would like to highlight the flexible nature of our software allowing the incorporation of additional rules that reflect current ideas on assembly (e.g. asymmetric rods with altered rules of assembly, incorporation of longitudinal movement during the post-accretion step and so forth). As such, we believe that the simulation environment developed here, represents a powerful framework on which more sophisticated models may be constructed and explored. Consequently we make our model freely available for public download (http://www.compsysbio.org/projects/rodDLA).

## Materials and Methods

### DLA of rod-like particles

Simulations and visualizations were performed using software developed in-house. The DLA simulator was written in C++ and was developed under Ubuntu Linux (version from 7.04–9.10). The 3D visualization tool was developed using QT (version 4.10 above) and OpenGL (http://www.opengl.org). All software is made freely available under the open source software license at http://www.compsysbio.org/projects/rodDLA.

DLA simulations were based on an unconstrained off-lattice based three dimensional environment. At the center of this environment was placed a single ‘seed’ rod. We decided to treat proteins as rigid rods in the current model largely to reduce the level of computational complexity. It is appreciated that there may be additional flexibility in the system that is not captured in this model which may preclude e.g. bending motions. However there is little information available on the types of additional motion that may be expected in our system. Furthermore, attempting to introduce such flexibility would significantly increase computational calculations precluding a global exploration of the parameters investigated here particularly for systems composed of hundreds to thousands of monomers. Since our main objective is to investigate the role that domain configuration may have on monomer alignment and how this may propagate to morphological and structural properties for aggregates composed of 1000's of monomers, we chose to begin with a relatively simple model. Rod diameters were fixed for all simulations at 1 unit while lengths varied depending upon simulation from 5 units to 34 units. Rod architectures were predefined at the beginning of each simulation. Simulations involved both symmetric and asymmetric rods and ranged from rods with a single elastomeric domain to rods with up to 40 alternating domains. In a typical DLA simulation, new rods are released one at a time from a random point on a release surface represented by a cylindrical surface in the middle with two spherical surfaces on the ends located 30 units from the growing aggregate (Figure 1A). After release rods perform a random walk in which, during each iteration, a direction is chosen at random and the rod is moved 0.5 units. The process is repeated until either the rod accretes to the growing aggregate (see below) or it moves 60 units from the center of the aggregate, in which event they are destroyed and a new rod is released. The process is repeated until 10,000 rods have accreted. As a simple model of the hydrophobic forces driving rod assembly, the probability of rod accretion is simply determined by the percentage of overlap of elastomeric domains. Overlap was defined as any overlap between the elastomeric domain of the incoming rod and the elastomeric domain of a neighbouring rod, using a distance threshold between rod axes of 1.2 units.

### Surface diffusion

After accretion, to further account for the contribution of interactions between neighbouring elastomeric domains to minimize surface exposure, rods were permitted to laterally diffuse over the surface of the aggregate. This was achieved by a series of putative rotations of up to 45 degrees, either clockwise or anti-clockwise, around the center of a randomly selected adjacent neighbour (direction and angle of rotation are randomly selected). To determine if the rotation is accepted, the total overlap of hydrophobic surface, using the distance threshold between rod axes of 1.2 units noted above, was calculated and used to assign a probability according to the formula based on the Boltzman function [62]:
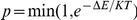
where ΔE represents the change in energy calculated as the proportion of exposed hydrophobic surface before and after the proposed move. *KT* is a tunable temperature term. The number of putative rotations is determined by the tunable parameter *X*. Diffusion of previously accreted rods was not allowed to reduce the level of computational complexity in the system. Again to reduce computational complexity, we did not allow surface diffusion along the long axis of the rod.

### Quantitative measurements

Aggregate *length* is simply the length in units of the aggregate on the longest dimension. The *connectivity* of a rod is defined as the number of neighbours of the rod. Neighbours are defined as rods whose central axes are within 1.2 units of each other. Aggregate *density* is defined as the number of rods that transverse a defined cross section of length 10 units along the longest axis of the aggregate. The cross section is defined as a convex hull that encompass all the rods that transverse it, computed using Graham's Scan algorithm [63]. *Surface area* is approximated through dividing the aggregate into 10 unit sections along the longest axis of the aggregate. Perimeters of each section are then summed. The s*urface area is* calculated only for the central section that occupies 50% of the aggregate's length. A *cross-link* between two rods is defined if the overlap between two neighbouring cross-linking domains is at least 50% of the length of both domains. Cross-linking networks are generated by treating rods as individual nodes. Edges between the nodes are then created if the rods share at least one cross-link. *Betweenness*, *cluster coefficients*, and *node degree* were computed using the BGL library in MatLab (http://www.mathworks.com/matlab).

## Supporting Information

Figure S1 **Impact of surface diffusion on aggregate morphology for a rod composed of multiple domains.** (A) View of a section of an aggregate generated with rods composed of an alternating domain architecture of two cross-linking domains (red) and three elastomeric domains (blue). Each domain was four units in length and the rod had a diameter of one unit. The aggregate was constructed using *KT* = 20 and *X* = 1,000. (B) Phase diagram showing a two dimensional cross-section through a central 60 unit section of representative aggregates composed of multi-domain rods grown under different values of *KT* and *X*. (C) Side views of 80 units of the central section of representative fibrils from (B). (D) Quantitative measures of morphological characteristics of aggregates represented in the phase diagram (B). Aggregates were grown using 10,000 rods, standard deviations are from 10 replicates.
(EPS)

Figure S2 **Quantitative measures of aggregate morphology and stability for 32 different rod architectures.** Graphs showing measures of morphology and stability for aggregates generated for 32 different rod architectures composed of different numbers and sizes of elastomeric and cross-linking domains. Domain architectures are indicated at the bottom and are ordered according to increasing average node degree (Figure 3). Error bars indicate standard deviations for ten replicates.
(EPS)
